# Prediction of acute coronary syndrome in patients with myeloproliferative neoplasms

**DOI:** 10.3389/fcvm.2024.1369701

**Published:** 2024-06-25

**Authors:** Jingfeng Huang, Ping Zhang, Fangjie Shen, Xiaodong Zheng, Qianjiang Ding, Yuning Pan, Xinzhong Ruan

**Affiliations:** ^1^ Department of Radiology, The First Affiliated Hospital of Ningbo University, Ningbo, China; ^2^ Department of Hematology, The First Affiliated Hospital of Ningbo University, Ningbo, China

**Keywords:** myeloproliferative neoplasms, acute coronary syndrome, coronary artery calcium score, predictive value, independent risk factors

## Abstract

**Background:**

Patients with myeloproliferative neoplasms (MPN) are exposed to a higher risk of cardiovascular disease, especially cardiovascular calcification. The present research aimed to analyze the clinical features and coronary artery calcium score (CACS) in MPN patients, and construct an effective model to predict acute coronary syndrome (ACS) in MPN patients.

**Materials and methods:**

A total of 175 MPN patients and 175 controls were recruited from the First Affiliated Hospital of Ningbo University. Based on cardiovascular events, the MPN patients were divided into the ACS group and the non-ACS group. Multivariate Cox analysis was completed to explore ACS-related factors. Furthermore, ROC curves were plotted to assess the predictive effect of CACS combined with white blood cells (WBC) and platelet for ACS in MPN patients.

**Results:**

The MPN group exhibited a higher CACS than the control group (133 vs. 55, *P* < 0.001). A total of 16 patients developed ACS in 175 MPN patients. Compared with non-ACS groups, significant differences in age, diabetes, smoking history, WBC, percentage of neutrophil, percentage of lymphocyte, neutrophil count, hemoglobin, hematocrit, platelet, lactate dehydrogenase, *β*_2_-microglobulin, and JAK2V617F mutation were observed in the ACS groups. In addition, the CACS in the ACS group was also significantly higher than that in the non-ACS group (374.5 vs. 121, *P* < 0.001). The multivariable Cox regression analysis identified WBC, platelet, and CACS as independent risk factors for ACS in MPN patients. Finally, ROC curves indicated that WBC, platelet, and CACS have a high predictive value for ACS in MPN patients (AUC = 0.890).

**Conclusion:**

CACS combined with WBC and platelet might be a promising model for predicting ACS occurrence in MPN patients.

## Introduction

Patients with myeloproliferative neoplasms (MPN) are characterized by clonal proliferation of hematopoietic stem cells, resulting in abnormal production of peripheral blood cells (1). MPN includes primary myelofibrosis (PMF), essential thrombocythemia (ET), and polycythemia vera (PV) (2). The pathogenesis of MPN is mainly caused by the mutations of JAK2, MPL, and CALR genes. In addition to the above three driver mutations, there are a variety of other mutations in DNA methylation-related genes, chromatin modification-related genes, and splicing complex-related genes (3, 4). According to the meticulous records maintained by the Norwegian Cancer Registry, the annual incidence of PV, ET, and PMF per 100,000 population significantly increased from 1995 to 1997 to 2010–2012 (from 0.4, 0.3, and 0.2 to 0.7, 1.0, and 0.5 per 100,000, respectively) (2). It can be seen that the incidence of MPN is increasing, which seriously endangers health.

Thromboembolism is the common cause of complication and death in MPN patients, manifesting as coronary heart disease (CHD), transient ischemic attack, cerebrovascular disease, and deep vein thrombosis (5). A meta-analysis of 13,436 MPN patients revealed an overall thrombosis rate of 20% in patients diagnosed with MPN (6). The related thrombotic complications of MPN patients mainly include arterial thrombosis, while the most common reason for death is acute coronary syndromes (ACS) (7). A Swedish study in patients with MPN from 1973 to 2005 reported a significant increase in cardiovascular mortality in young MPN patients during the first decade after diagnosis (8). Therefore, evaluating the risk of ACS in MPN patients in advance holds great significance, allowing early intervention and potentially improving prognosis.

The pathologic mechanism of thrombosis in MPN patients is intricate and is mainly affected by disease-related factors such as JAK2 gene mutation and cytosis (9). MPN is characterized by a significant increase in blood cells, which results in an increased risk of thrombus formation (10). The study with 142 ET patients complicated with thrombosis showed that JAK2V617F positivity was an independent risk factor for recurrent thrombosis in ET patients (11). However, the mechanism of thrombosis in JAK2V617F-positive patients has not been fully elucidated, and it is believed that JAK2V617F-positive patients often have higher blood cell count levels (12). The non-disease-related factors mainly include age, previous history of thrombosis, cardiovascular risk factors (such as hyperlipidemia, smoking history, diabetes, and hypertension), oral contraceptives, and pregnancy. Although current risk stratification methods could help assess the risk of thrombosis, some MPN patients are still inaccurately evaluated (13). For instance, traditional thrombus prediction systems for PV and ET are mainly based on the history of thrombosis and age (14). Patients older than 60 years or with a thrombosis history were classified into the high-risk group (15). However, these evaluation systems do not accurately determine the risk of thrombosis in MPN patients, especially for ACS, which still occurs in some low-risk patients (16). Therefore, relevant factors should be further explored to provide a good model with increased accuracy for ACS prediction.

Coronary artery calcification (CAC) is a common characteristic of advanced atherosclerosis, which can reflect the presence and severity of CHD (17). Coronary artery calcium score (CACS) is an evaluation index of CAC, which is widely used in clinics to predict cardiovascular events (18–20). Patients with high CACS are positively correlated with the incidence of coronary artery stenosis and major adverse cardiovascular events (21). A previous study reported that in MPN patients, the prevalence of CACS > 400 in coronary arteries and aortic valve calcification was significantly higher, suggesting an association between MPN patients and the higher risk of cardiac calcification (22). However, whether CACS can predict ACS in MPN patients remains unknown.

In the present study, the differences in clinical characteristics and CACS between MPN patients and normal controls were retrospectively analyzed. Moreover, the risk factors associated with ACS in MPN patients were explored. Based on these risk factors, a new model was constructed to predict the occurrence of ACS.

## Materials and methods

### Patients

We recruited 175 MPN patients and 175 controls from The First Affiliated Hospital of Ningbo University from January 2018 to January 2020. The MPN patients were diagnosed according to the World Health Organization, and all the patients were above 18 years old with complete clinical data. The exclusion criteria were: (1) acute infection; (2) combined with other malignant tumors; (3) patients who terminated follow-up due to transfer or other reasons. In addition, the 175 non-MPN patients were matched to the MPN patients based on age and sex. The ethics committee approved this retrospective study (approval No. 2022-022A).

### Clinical characteristics

General information, including age, sex, splenomegaly, past thrombosis history, smoking history, duration of the disease, hypertension, hyperlipidemia, diabetes mellitus, cytoreductive therapy, and other treatments were obtained from the medical records. In addition, blood test data from the first hospitalization, including red blood cells, hematocrit, hemoglobin, glucose, white blood cells (WBC), percentage of neutrophil, percentage of lymphocyte, neutrophil count, lymphocyte count, red blood cell distribution width, platelet, total cholesterol, triglyceride, erythrocyte sedimentation rate (ESR), low-density lipoprotein cholesterol (LDLC), D-dimer levels, *β*_2_-microglobulin (*β*_2_-MG), high-density lipoprotein cholesterol (HDLC) and lactate dehydrogenase (LDH) were also recorded. The results of gene mutation detection including JAK2, CARL, and MPL were collected in the MPN patients. Furthermore, the ACS events during the 3-year observation period following standard treatment for MPN patients were recorded. The diagnosis for ACS was according to the ACC/AHA Guidelines 2016 (23).

### Coronary artery calcification score

Cardiac CT was performed on a 320-slice CT Scanner (Aquilion ONETM, Toshiba, Japan) from the first hospitalization at the time of diagnosis of MPN. The images were submitted to the relevant staff and the calcification scores of the left main coronary artery (LMA), right coronary artery (RCA), left circumflex artery (LCX) and left anterior descending artery (LAD) were calculated. Areas with a density >130 Hounsfield units were automatically identified. The calculation of calcification was as follows: >400 Hu scored 4 points, 300–399 Hu scored 3 points, 200–299 Hu scored 2 points, and 130–199 Hu scored 1 point. The calcification integral was calculated by the following formula: Calcification Integral = calcification area × CT score. CACS is obtained by adding all the calcification integrals together. Subsequently, patients with a CACS 0–100 score were incorporated into the low-risk group for cardiovascular events, those with a score of 100–300 were assigned to the intermediate-risk group, and those with a score >300 were assigned to the high-risk group (24).

### Statistical analysis

Statistical analysis was performed using the SPSS software (version 23.0, IBM). Continuous variables are presented as mean ± SD if normally distributed or as median with interquartile range (IQR) otherwise. Categorical variables are presented as the number and percentage of patients. The *t*-test or Wilcoxon rank sum test was used for comparison. We used multivariate Cox regression analysis to analyze ACS-related factors. ROC curves were drawn to evaluate the accuracy of CACS combined with WBC and platelets in the prediction of ACS in MPN patients. Statistical significance was indicated by two-sided *P* < 0.05.

## Results

### The clinical characteristics of MPN and controls

We enrolled 220 patients in this study and 45 patients were excluded according to the criteria. Consequently, 175 patients (mean age 54.79 years ± 14.70, 60% men) were finally included. PV accounted for nearly half of the MPNs (86 cases, 49.1%), followed by ET (68 cases, 38.9%) and PMF (21 cases, 12.0%). The 175 MPN patients were defined as the MPN group and the 175 normal physical examination patients were matched by sex and age as the control group.

Statistically significant differences in hypertension, diabetes, hyperlipidemia, splenomegaly, past thrombosis history, smoking history, red blood cell, hematocrit, red cell distribution width, hemoglobin, WBC, platelet, ESR, triglyceride, total cholesterol, LDLC, LDH, *β*_2_-MG, and D-dimer levels were observed between the control group and MPN group (Table 1).

**Table 1:** The clinical characteristics of the MPN group and the control group.

Characteristic	MPN group (*n* = 175)	Control group (*n* = 175)	*P* value
Age (years)	54.79 ± 14.70	57.61 ± 13.04	0.059
Male (%)	105 (60.0)	105 (60.0)	1.000
Hypertension (%)	62 (35.4)	38 (21.7)	0.005
Diabetes (%)	26 (14.9)	11 (6.3)	0.009
Hyperlipidemia (%)	40 (22.9)	25 (14.3)	0.039
Splenomegaly (%)	96 (54.9)	2 (1.1)	<0.001
Past thrombosis history (%)	20 (11.4)	3 (1.7)	<0.001
Smoking (%)	54 (30.9)	37 (26.8)	0.038
White blood cell (*10^9 ^/L)	8.83 ± 6.51	6.12 ± 2.00	<0.001
Red blood cell (*10^12 ^/L)	4.76 ± 1.55	4.27 ± 0.54	<0.001
Hemoglobin (g/L)	139.67 ± 44.03	129.12 ± 17.80	<0.001
Hct (%)	43.57 ± 12.50	39.11 ± 4.97	<0.001
RDW (%)	15.36 ± 3.47	12.69 ± 1.15	<0.001
Platelet (*10^9 ^/L)	522.96 ± 415.52	224.19 ± 64.69	<0.001
ESR (mm/H)	19 (10–42)	21 (12–31)	0.03
Glucose (mmol/L)	5.01 ± 1.49	5.21 ± 1.02	0.148
Triglyceride (mmol/L)	1.64 ± 1.00	1.37 ± 0.81	0.008
Total cholesterol (mmol/L)	4.14 ± 1.21	4.65 ± 1.06	<0.001
HDLC (mmol/L)	1.41 ± 1.39	1.26 ± 0.40	0.188
LDLC (mmol/L)	2.71 ± 0.98	3.09 ± 0.71	<0.001
LDH (U/L)	325.06 ± 334.63	181.48 ± 39.52	<0.001
*β*_2_-MG (mg/L)	2.41 ± 1.49	1.83 ± 0.81	<0.001
D-dimer (ng/ml)	167 (104–355)	123 (55–167)	<0.001

Hct, hematocrit; RDW, red cell distribution width; ESR, erythrocyte sedimentation rate; HDLC, high-density lipoprotein cholesterol; LDLC, low-density lipoprotein cholesterol; LDH, lactate dehydrogenase; *β*_2_-MG, *β*_2_-microglobulin.

### High CACS scores in the MPN patients

**Figure 1:**
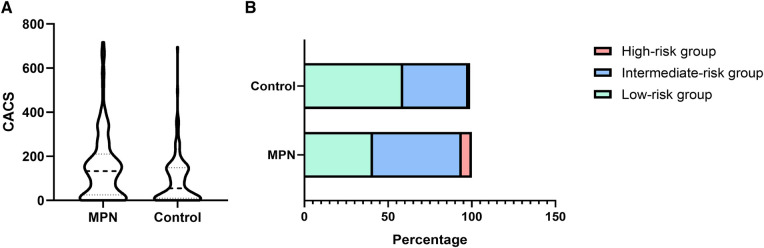
The difference in CACS (**A**) and proportion (**B**) between the MPN and control groups.

Compared with the controls, the CACS in the MPN group was significantly higher (133 (IQR 25–210) vs. 55 (IQR 10–148), Figure 1A, *P* < 0.001). Besides, the proportion of the low-risk, intermediate-risk, and high-risk groups in MPN and controls were significantly different (59.4% vs. 41.1%, 36.0% vs. 44.0%, and 4.6% vs. 14.9%, *P* < 0.001, respectively). The proportion of the low-risk group was relatively high in the control group. In contrast, the proportions of the intermediate-risk group and high-risk group in the MPN group were significantly higher (Figure 1B).

### The ACS events in MPN patients

The occurrence of ACS was analyzed both in the MPN group and the control group. In the MPN group, 16 (9.1%) patients developed ACS within 3 years, including 11 cases of STEMI, 3 cases of unstable angina pectoris, and 2 cases of NSTEMI. Among these 16 MPN patients who developed ACS, 10 patients were diagnosed with PV, including 7 cases of STEMI, 2 cases of unstable angina pectoris, and 1 case of NSTEMI. In addition, 5 ET patients developed ACS, including 4 cases of STEMI and 1 case of NSTEMI. One PMF patient developed ACS in the form of unstable angina pectoris. However, only 2 patients (1.1%) developed ACS in the control group, including 1 case of unstable angina pectoris and 1 case of STEMI.

### The clinical characteristics of ACS patients

The 16 MPN patients who developed ACS were assigned to the ACS group, and the 159 MPN patients who did not develop ACS were assigned to the non-ACS group. The ACS group was older. Additionally, diabetes, smoking history, WBC, percentage of neutrophil, percentage of lymphocyte, neutrophil count, hemoglobin, hematocrit, platelets, LDH, *β*_2_-MG and gene mutation of JAK2V617F were observed in the ACS group. The rate of JAK2V617F mutation in the ACS group and non-ACS group was significantly different (100.0% vs. 59.1%, *P* < 0.001). Besides, one case had CALR and JAK2V617F double mutations, and another case had MPL and JAK2V617F double mutations in the ACS group. There were no significant difference in cytoreductive therapy, anticoagulant therapy, antihypertensive therapy, antilipemic therapy, and antidiabetic therapy between the two groups (Table 2).

**Table 2:** Comparison of clinical characteristics between the ACS group and the non-ACS group of MPN patients.

Characteristic	ACS group (*n* = 16)	Non-ACS group (*n* = 159)	*P* value
Age (years)	67.50 ± 10.74	53.52 ± 14.46	<0.001
Male (%)	10 (62.5)	95 (59.7)	1.000
Hypertension (%)	8 (50.0)	54 (34.0)	0.272
Diabetes (%)	6 (37.5)	20 (12.6)	0.017
Hyperlipidemia (%)	6 (37.5)	34 (21.4)	0.207
Splenomegaly (%)	8 (50.0)	88 (55.4)	0.794
Past thrombosis history (%)	3 (18.8)	14 (8.8)	0.217
Smoking (%)	10 (62.5)	44 (27.7)	0.008
Duration of disease (month)	75.6 ± 38.5	71.5 ± 46.4	0.733
White blood cell (*10^9 ^/L)	18.86 ± 6.51	7.82 ± 4.18	<0.001
Percentage of neutrophil (%)	76.33 ± 9.08	65.34 ± 15.92	0.007
Percentage of lymphocyte (%)	12.71 ± 5.49	24.12 ± 13.56	0.001
Neutrophil count (*10^9 ^/L)	14.98 ± 11.04	6.81 ± 12.07	0.010
Lymphocyte count (*10^9 ^/L)	1.93 ± 0.94	1.87 ± 2.01	0.902
Red blood cell (*10^12 ^/L)	4.23 ± 1.63	4.81 ± 1.54	0.153
Hemoglobin (g/L)	117.31 ± 39.27	141.92 ± 43.96	0.033
Hct (%)	44.17 ± 12.48	37.60 ± 11.43	0.045
RDW (%)	16.27 ± 4.19	15.26 ± 3.39	0.271
Platelet (*10^9 ^/L)	697.50 ± 411.25	505.40 ± 413.14	0.048
ESR (mm/H)	25 (0.25–60.5)	19 (10–41)	0.323
Glucose (mmol/L)	4.91 ± 1.93	5.03 ± 1.45	0.759
Triglyceride (mmol/L)	1.38 ± 0.56	1.66 ± 1.03	0.279
Total cholesterol (mmol/L)	3.65 ± 1.26	4.19 ± 1.19	0.093
HDLC (mmol/L)	1.12 ± 0.34	1.44 ± 1.45	0.385
LDLC (mmol/L)	2.40 ± 0.86	2.74 ± 0.98	0.189
LDH (U/L)	480.69 ± 263.98	309.40 ± 337.64	<0.001
*β*_2_-MG (mg/L)	3.96 ± 3.02	2.25 ± 1.14	<0.001
D-dimer (ng/ml)	167 (106–360)	161 (99–333.75)	0.723
Driver gene mutation rate			
JAK2V617F (%)	16 (100)	94 (59.1)	0.001
CALR (%)	1 (6.25)	27 (17.0)	0.474
MPL (%)	1 (6.25)	11 (6.9)	1.000
Cytoreductive therapy			
Interferon (%)	5 (31.3)	89 (56.0)	0.069
Hydroxycarbamide (%)	10 (62.5)	67 (42.1)	0.185
Ruxolitinib (%)	1 (6.3)	3 (1.9)	0.321
Other treatments			
Anticoagulant therapy (%)	14 (87.5)	100 (62.9)	0.056
Antihypertensive therapy (%)	8 (50.0)	60 (37.7)	0.421
Hypoglycemic therapy (%)	6 (37.5)	29 (18.2)	0.095
Hypolipidemic therapy (%)	6(37.5)	26(16.4)	0.081

Hct, hematocrit; RDW, red cell distribution width; ESR, erythrocyte sedimentation rate; HDLC, high-density lipoprotein cholesterol; LDLC, low-density lipoprotein cholesterol; LDH, lactate dehydrogenase; *β*_2_-MG, *β*_2_-microglobulin.

### High CACS scores in the MPN patients with ACS

**Figure 2:**
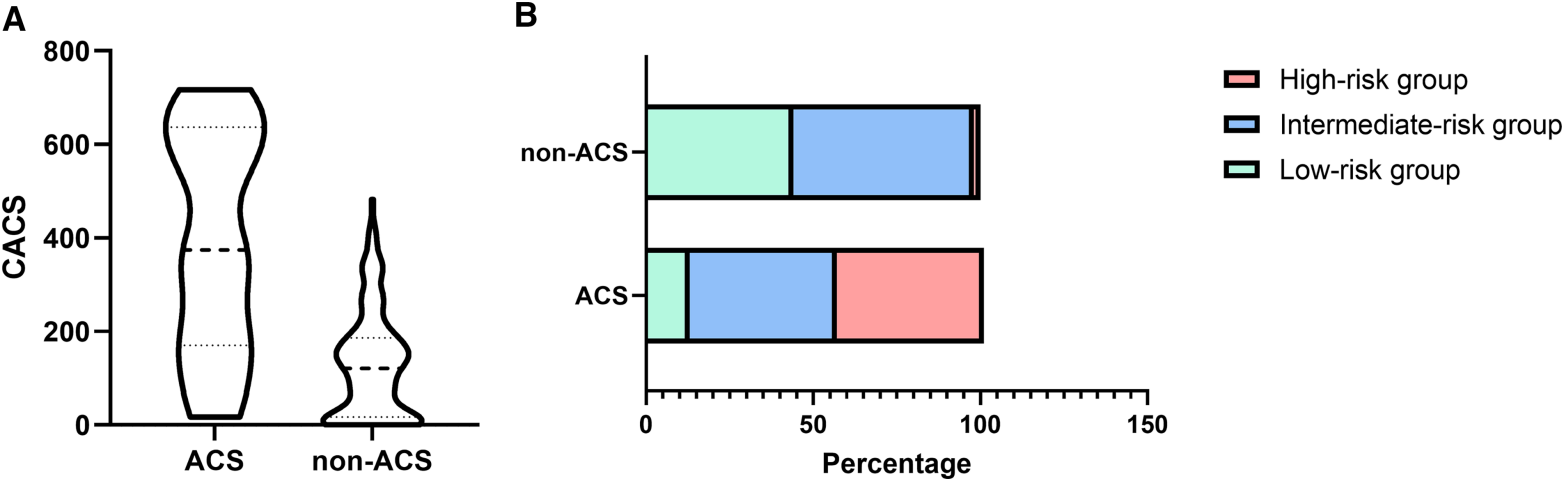
The difference in CACS (**A**) and proportion (**B**) between the ACS and non-ACS groups of MPN patients.

Compared with the non-ACS group, a significantly higher median CACS was observed in the ACS group (374.5 (IQR 170.5–636.75) vs. 121 (IQR 17–186), Figure 2A, *P* < 0.001). Furthermore, patients in the ACS group were mostly in the intermediate-risk and high-risk groups, while patients in the non-ACS group were mainly in the low-risk group (Figure 2B). Therefore, it is highly likely that high CACS is associated with the occurrence of ACS in MPN patients.

### The risk factors and prediction of ACS in MPN patients

Subsequently, the traditional risk factors and factors with significant differences described above were included in the Cox regression analysis. The univariate analysis showed that age, smoking, WBC, percentage of neutrophil, neutrophil count, hemoglobin, Hct, platelet, LDH, CACS and gene mutation of JAK2V617F might be independent risk factors for ACS. The multivariate Cox regression analysis suggested that platelet [Hazard Ratio (HR) = 1.108, 95% CI: 1.018–1.207; *P* = 0.018], WBC (HR = 1.092, 95% CI: 1.047–1.139; *P* < 0.001), and CACS (HR = 1.005, 95% CI: 1.002–1.008; *P* = 0.001) were independent risk factors for ACS in MPN patients (Table 3).

**Table 3:** Multivariate cox regression analysis of ACS incidence in MPN patients.

Characteristic	Univariate analysis			Multivariable analysis		
	HR	95% CI	*P* value	HR	95% CI	*P* value
Age	1.095	1.036–1.158	0.001	1.012	0.931–1.102	0.773
Gender	1.148	0.412–1.196	0.792	–	–	–
Hypertension	0.672	0.193–1.341	0.532	–	–	–
Diabetes	1.234	0.736–1.788	0.156	–	–	–
Hyperlipidemia	1.352	0.458–1.950	0.585	–	–	–
Splenomegaly	0.653	0.231–1.845	0.422	–	–	–
Smoking	1.034	1.008–1.059	0.039	1.470	0.079–1.789	0.406
WBC	1.078	1.041–1.115	<0.001	1.092	1.047–1.139	<0.001
Percentage of neutrophil	1.065	1.015–1.116	0.009	1.007	1.004–1.009	0.490
Neutrophil count	1.022	1.004–1.041	0.018	1.007	1.005–1.010	0.554
Hemoglobin	1.033	1.018–1.048	<0.001	1.066	0.979–1.162	0.142
Hct	1.001	1.000–1.003	0.030	0.774	0.574–1.043	0.092
Platelet	1.155	1.085–1.229	<0.001	1.108	1.018–1.207	0.018
LDH	1.001	1.000–1.001	0.045	0.998	0.995–1.001	0.128
CACS	1.007	1.004–1.009	<0.001	1.005	1.002–1.008	0.001
JAK2V617F	1.035	1.034–1.037	0.037	1.093	1.048–1.139	0.493

WBC, white blood cell; Hct, hematocrit; LDH, lactate dehydrogenase.

**Figure 3:**
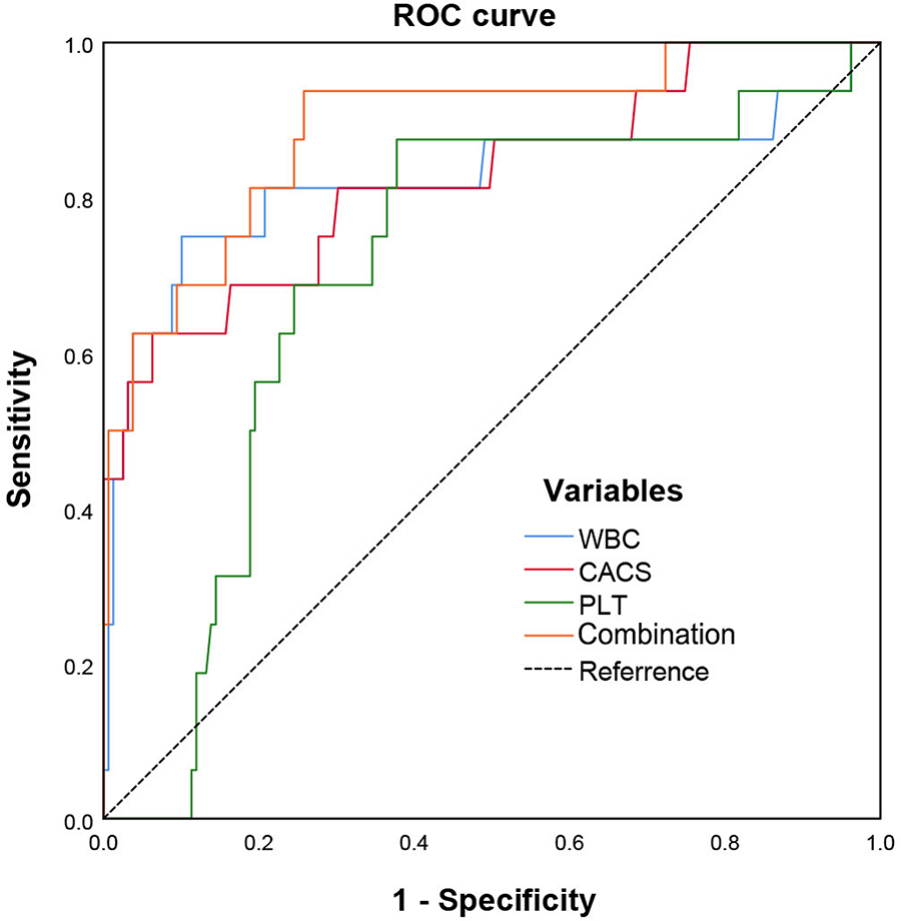
ROC curves of CACS combined with WBC and platelet in predicting acute coronary syndrome in MPN patients.

As shown in Figure 3, the area under curve (AUC) of ACS evaluated by WBC was 0.821, with 75.0% sensitivity, and 89.9% specificity (the cutoff value was 11.65); by platelet was 0.704, with 87.5% sensitivity, 62.3% specificity (the cutoff value of 604); by CACS was 0.826, with 62.5% sensitivity, 93.7% specificity (the cutoff value of 334). Notably, the combination of three factors could increase the potential to predict ACS with an AUC of 0.890, a sensitivity of 93.8%, and a specificity of 74.2%.

## Discussion

Arterial thrombosis, the direct cause of cardiovascular events, is the main risk factor affecting the survival of MPN patients (25). Thromboembolism-related mortality accounts for 35%–70% of the total mortality of MPN (26), with ACS being the common one (27). Therefore, early identification and timely intervention of high-risk patients may significantly reduce the incidence of ACS in MPN patients. Our study showed a significant increase in CACS in MPN patients, indicating severe coronary artery calcification. In addition, through multivariate Cox analysis, WBC, platelet, and CACS were confirmed to be independent risk factors for ACS in MPN patients. Hence, a new model was developed based on CACS combined with WBC and platelet to predict ACS in MPN patients. The sensitivity and specificity in predicting ACS in MPN patients were high. These findings provide a theoretical basis for establishing a promising prediction model for early prediction of ACS risk.

Previous studies showed that age, gender, diabetes and hyperlipidemia were independent risk factors for cardiovascular disease in MPN patients (28–31). Furthermore, a prospective study revealed that hypertension was a risk factor for future arterial thrombosis, while age 65 or higher was a predictor of future venous thrombosis (32). In the current research, traditional risk factors for cardiovascular disease, including age, smoking history, and hyperglycemia were different between ACS and non-ACS. Consequently, traditional risk factors cannot be ignored in MPN patients.

In addition to the factors mentioned above, multivariate Cox analysis confirmed that CACS, WBC, and platelet were independent risk factors for ACS. At present, whether WBC is an independent risk factor for ACS in MPN patients remains controversial. Some studies reported that WBC could not predict thrombotic events in MPN patients (33, 34). However, other studies showed that leukocytosis might be a risk factor for thrombosis (35–39). For instance, the ECLAP study indicated that leukocytosis was an independent risk factor for the development of arterial thrombosis, which was significantly increased in patients with WBC > 15*10^9^/L (9). In this study, WBC levels were significantly higher in the MPN group, and also higher in the ACS group. Due to inflammatory response, high WBC in MPN patients may lead to arterial thrombosis and ACS. Wolach O et al. showed that mice with conditional knock-in of the JAK2V617F gene have an increased propensity to form neutrophil extracellular trap, which is a component of innate immunity associated with thrombosis (40). Besides, JAK2V617F mutation in a murine MPN model could alter vascular endothelial function, making it more affinity and prothrombotic, thereby promoting MPN-related cardiovascular complications (41). The specific mechanism is worthy of further study in the future.

Platelet plays an indispensable part in thrombosis, and the increased platelet adhesion and activation are associated with thrombus formation. A previous study reported that platelet >1,000*10^9 ^/L at initial diagnosis was related to a lower risk of arterial thrombosis (42). Interestingly, the high platelet in ET patients was more likely to result in thrombotic events when the patients had a normal WBC count (43). Besides, severe thrombocytosis (>1,500*10^9 ^/L) in acquired von Willebrand disease might increase the risk of bleeding (44). Our study showed that ACS patients had higher platelet levels than non-ACS patients. Notably, the average platelet level of MPN patients in this study was 522.96*10^9^/L, which was much lower than in the above studies. Nevertheless, our findings were limited by the small size of patients, so large sample prospective studies should be conducted to verify the role of platelet in MPN patients.

CACS could effectively quantify coronary calcification and degree, which was a suitable indicator for coronary heart disease (45). Hecht et al. analyzed five major multi-center studies and determined the 10-year risk of cardiovascular events based on the CACS. For a CACS of 1–100, the risk of cardiovascular events was 1%–10%, for a CACS of 100–400, the risk of cardiovascular events was 11%–20%, and when the CACS > 400, the risk of cardiovascular events was higher than 20% (46, 47). The MESA study revealed that the incidence of MACE (including recurrent angina pectoris, acute myocardial infarction, severe arrhythmia, heart failure, and death from coronary heart disease) in the CACS 1–100, 101–300, and >300 groups were 3.16, 7.73, and 9.67 times higher than that in controls, respectively (48). In a previous study, a significantly higher proportion of patients with a CACS of >160 was observed in the ET group, suggesting that the risk of atherothrombotic events was increased in MPN patients (49). In this study, MPN patients were assigned to low, intermediate, and high-risk groups for cardiovascular events based on CACS. The CACS of MPN patients was higher than that of controls, with a higher proportion of intermediate and high-risk patients, indicating that MPN patients had more obvious coronary artery calcification. Our study showed that the risk of ACS was 38.5% in the high-risk group, 5.2% in the intermediate-risk group, and 2.8% in the low-risk group. In summary, CACS may be a potential predictor of ACS in MPN patients.

Currently, multiple models have been proposed to predict cardiovascular events in different kinds of diseases. In our previous study, the combination of vertebral bone mineral density and CACS could predict the incidence of cardiovascular events in maintenance hemodialysis patients (50). In addition, NT-proBNP was identified to be an indicator with a high predictive value for the risk of cardiovascular diseases in ACS patients with oncological diseases (51). The AUC of our model was 0.890 with a high sensitivity and specificity in predicting ACS in MPN patients. These results suggested that the model had good predictive value for the risk of ACS in MPN patients. Additionally, CACS, WBC, and platelets were ACS-related risk factors worthy of further investigation in MPN patients.

JAK2, CALR, and MPL gene mutations were also detected in 175 MPN patients. The frequency of JAK2, CALR, and MPL mutations in this study was 62.9% (110/175), 16.0% (28/175), and 6.9% (12/175), respectively. In addition, the JAK2 V617F gene mutations were significantly higher in the ACS group than in the non-ACS group. Barbui et al. found that JAK2 V617F mutation-positive PV patients have higher WBC and hemoglobin, and are more likely to have thrombotic events than JAK2 V617F mutation-negative patients (44). In ET patients, those with JAK2 gene mutation positive had higher WBC and hemoglobin and were more prone to thrombotic events (52). Therefore, JAK2 V617F mutation might be associated with a higher incidence of thrombotic events.

Notably, 1 case had CALR and JAK2 V617F double mutations and another patient had MPL and JAK2 V617F double mutations in the ACS group. These findings contradict the hypothesis that mutations in JAK2, CALR, and MPL are mutually exclusive (53). Although these co-mutations are rare in MPN patients, there have been a few reports (54, 55). For instance, patients with CALR and JAK2 gene co-mutations were found in Colombian MPN patients (56). Min-Gu Kang et al. found that JAK2 and CALR mutations coexisted in 7 (4.2%) of 167 ET patients (57). However, the current evidence cannot confirm whether the presence of co-mutations might affect the occurrence of thrombotic events and ACS, and future cohort studies are worth conducting to answer this question.

In addition to disease driver mutations, clonal hematopoiesis of indeterminate potential (CHIP) was also associated with CHD. CHIP increases coronary artery calcification, which is a marker of coronary atherosclerotic burden. Jaiswal et al. found that carriers of CHIP had a 1.9-fold increased risk of CHD compared to non-carriers (58). Besides, Zhao et al. found that CHIP not only significantly increases the risk of CHD, but also shows a significant additive effect with the genetic risk of innate inflammation (59). CHIP and chronic inflammation are typical features of MPN, and CHIP can promote inflammatory response. Furthermore, chronic inflammation is the main driver of early atherosclerosis and disease progression in MPN patients (60). Therefore, there is a close relationship between CHIP, CHD, and MPN. In the future, it is expected that more research will be devoted to the mechanisms of CHIP and CHD in MPN.

Nonetheless, the limitations should be acknowledged. Although PV, ET, and PMF are the MPN, their clinical symptoms are different. Due to the limited sample size, we did not explore them separately for the time being. The differences between subtypes may warrant further discussion. Furthermore, the constituent ratios of patients with PV, ET, and MPL were 62.5%, 31.3%, and 6.3%, respectively in the ACS group. In the non-ACS group, the constituent ratios were 47.8%, 39.6%, and 12.6%, respectively. The difference in the proportion of subtypes between the two groups and the relatively small total sample size may be why JAK2 is not an independent risk factor for ACS. In the future, a multi-center study with more indicators and a large sample size should be conducted to establish a more comprehensive and accurate thrombosis risk prediction model.

## Conclusion

In conclusion, CACS was significantly elevated in MPN patients, suggesting that MPN may increase the burden of coronary calcification. The CACS in MPN patients with ACS is significantly higher than that in MPN patients without ACS. CACS combined with WBC and platelets may provide an effective model to predict the risk of ACS in MPN patients.

## Data Availability

The raw data supporting the conclusions of this article will be made available by the authors, without undue reservation.
